# The Therapeutic Potential of *Laurus nobilis* L. Leaves Ethanolic Extract in Cancer Therapy

**DOI:** 10.3390/molecules30194012

**Published:** 2025-10-07

**Authors:** Farah Al-Mammori, Ashraf M. A. Qasem, Deniz Al-Tawalbeh, Duaa Abuarqoub, Ali Hmedat

**Affiliations:** 1 Department of Pharmaceutical Sciences, Faculty of Pharmacy, Zarqa University, Zarqa 13110, Jordan; fmamoori@zu.edu.jo (F.A.-M.); aqasem@zu.edu.jo (A.M.A.Q.); 2 Department of Medicinal Chemistry and Pharmacognosy, Faculty of Pharmacy, Yarmouk University, Irbid 21163, Jordan; deniz.altawalbeh@yu.edu.jo; 3 Department of Pharmacology and Biomedical Sciences, Faculty of Pharmacy and Medical Sciences, University of Petra, Amman 11196, Jordan; duaa.abuarqoub@uop.edu.jo; 4 Cell Therapy Center, The University of Jordan, Amman 11942, Jordan; 5 Department of Pharmaceutical Technology and Pharmaceutics, Faculty of Pharmacy, Yarmouk University, Irbid 21163, Jordan

**Keywords:** *Laurus nobilis*, anticancer, antioxidant, breast cancer, ovarian cancer, colorectal cancer, head and neck cancer, apoptosis

## Abstract

This study explores the anticancer, antioxidant, and phytochemical activities of *Laurus nobilis* L. ethanolic leaf extract. The extract demonstrated selective cytotoxicity against four human cancer cell lines, showing strong cytotoxic effect against ovarian (ES2), head and neck (SAS), and colorectal (HT-29) cancer cells, with IC_50_ values ranging from 3.8 ± 0.3 to 4.4 ± 0.6 µg/mL. Notably, it exhibited only moderate inhibition of the MDA-MB-231 breast cancer cell line (IC_50_ = 18.5 ± 0.8 µg/mL), possibly reflecting intrinsic differences in cell line sensitivity. Importantly, the extract showed low toxicity toward normal human fibroblasts (HDF), with an IC_50_ value exceeding 100 µg/mL, indicating a favorable selectivity profile. The flow cytometry analysis showed that the extract caused cell death and stopped the cell cycle in both SAS and ES2 cancer cell lines. In SAS cells, extract treatment significantly increased apoptotic cells (21.1% ± 0.3%) compared to the control (6.3% ± 0.4%), along with G2 phase accumulation, indicating G2 arrest. Similarly, in ES2 cells, apoptosis increased (16.2% ± 1.3% vs. control 8.1% ± 1.0%), and a significant cell accumulation in the S phase was observed, suggesting disruption of cell cycle progression. Antioxidant screenings showed impressive dose-dependent DPPH radical scavenging activity (25–2000 µg/mL), although less potent than ascorbic acid (2.6 µg/mL). UPLC-QTOF/MS phytochemical analysis revealed various phenolic constituents, such as flavonoids and phenolic acids, and an inferred association with the recorded bioactivities. This preliminary work indicates that *L. nobilis* extracts may act as natural anticancer and antioxidant agents; however, it was limited to *in vitro* testing with non-standardized samples, underscoring the need for further research to validate and extend these findings for future applications.

## 1. Introduction

Cancer is the second-leading cause of death worldwide, and the global cancer burden is predicted to grow over the next two decades [1]. Conventional cancer treatments, such as chemotherapy, can impose significant physical and psychological burdens, prompting increased interest in plant-based remedies as safer, adjunctive options [2]. Throughout history, people have turned to nature as a source of medicine, a tradition that persists in modern healthcare. The global popularity of herbal medicine has grown significantly, particularly as complementary and alternative therapies gain attention for their potential role in managing diseases, including cancer [3,4].

*Laurus nobilis* (family Lauraceae), commonly known as bay leaf, is a well-known medicinal plant widely used in Mediterranean regions, including Jordan. Its leaves and fruits are valued for their aromatic properties and have traditionally been used to treat gastrointestinal issues such as bloating and indigestion [5]. The essential oil extracted from the leaves has been shown to have antibacterial, antioxidant, and anti-inflammatory properties. Additional leaves of *L. nobilis* have been used to cure rheumatism, neuralgia, and scabies [6]. The studies suggest that *L. nobilis* possesses phenolic compounds, including flavonoids, phenolic acids, and tannins (proanthocyanidins) [7], with antioxidant properties, potentially contributing to its anticancer effects [5]. Because oxidative stress is a well-documented phenomenon in cancer, it is reasonable that antioxidants can greatly reduce cancer incidence and development [8]. However, further research is required to validate its therapeutic applications and understand its mechanisms of action. In Jordan, breast cancer is the most common cancer among women and is often diagnosed at advanced stages and younger ages than in Western countries [9]. Ovarian cancer ranks as the sixth leading cause of cancer-related deaths among women globally [10], while colorectal cancer is the most prevalent cancer in Jordanian men and the second most common in women [8]. Additionally, head and neck cancers, the seventh most common malignancies worldwide, contribute significantly to male cancer mortality [11]. Despite the growing global interest in herbal medicine and preliminary reports highlighting the antioxidant and potential anticancer properties of *L. nobilis*, evidence for its efficacy against specific cancer types remains limited, particularly those prevalent in Jordan. This study is novel in demonstrating its selective activity against multiple cancer cell lines, including SAS head and neck cancer cells, while confirming safety on normal fibroblasts and revealing mechanisms such as apoptosis and cell cycle arrest. Therefore, the present work aims to evaluate the anticancer and antioxidant activities of the ethanolic leaf extracts of *L. nobilis* and to identify their main phytochemical constituents using UPLC-QTOF/MS. The study is preliminary and seeks to establish the potential use of *L. nobilis* extract components in the future.

## 2. Results and Discussion

### 2.1. Cytotoxic Activity

**Figure 1:**
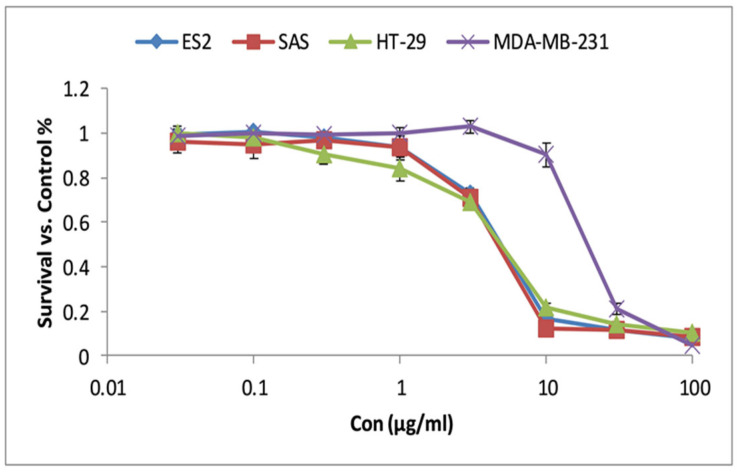
Cytotoxic activity of *L. nobilis* leaves ethanolic extract, as determined by the MTT assay. Dose–response curves show a concentration-dependent decrease in cell viability. Data are presented as mean ± SD from three independent experiments.

The MTT assay was performed to evaluate the anticancer activity of the extract against MDA-MB-231, SAS, HT-29, and ES2 cell lines. As shown in Figure 1, treatment with increasing concentrations of the extract (0.03–100 µg/mL) resulted in a dose-dependent decrease in cell viability, indicating its potential cytotoxic effect across all tested cell lines.

To quantify this effect, IC_50_ values were determined from dose–response curves, including normal human fibroblasts, to evaluate the extract’s selectivity toward cancer cells. As summarized in Table 1, the extract exhibited potent inhibition on ES2 (IC_50_ = 4.2 ± 0.2 µg/mL), SAS (IC_50_ = 3.8 ± 0.2 µg/mL), and HT-29 (IC_50_ = 4.4 ± 0.6 µg/mL) cell lines, while demonstrating moderate inhibition against MDA-MB-231 cells (IC_50_ = 18.5 ± 0.8 µg/mL). The noticeably higher IC_50_ value observed in MDA-MB-231 cells (18.5 µg/mL) compared to the values obtained for ES2, HT-29, and SAS cells (3.8–4.4 µg/mL) may reflect intrinsic differences in cell line sensitivity. MDA-MB-231 is a triple-negative breast cancer cell line known for its aggressive phenotype, high metastatic potential, and resistance to a wide range of chemotherapeutic agents, which may contribute to the reduced sensitivity observed in our assay [12]. This highlights the potential cell type-specific activity of the extract, which may be more effective against certain tumor types. Notably, the extract displayed low toxicity toward normal fibroblast cells (IC_50_ > 100 µg/mL), indicating selectivity for cancer cells.

To benchmark the anticancer efficacy of *L. nobilis* ethanolic extract, its IC_50_ values were compared with those of cisplatin, a widely used chemotherapeutic agent. As shown in Table 1, the extract exhibited comparable cytotoxicity to cisplatin in ES2 and SAS cells, with IC_50_ values of 4.2 ± 0.2 and 3.8 ± 0.2 µg/mL, respectively, versus 3.6 ± 0.3 and 2.9 ± 0.5 µg/mL for cisplatin. Notably, the extract demonstrated superior cytotoxicity in HT-29 colorectal cancer cells (4.4 ± 0.6 µg/mL) compared to cisplatin (10.7 ± 2.1 µg/mL), underscoring its potential therapeutic relevance in colorectal cancer. For MDA-MB-231, a triple-negative breast cancer cell line known for its chemoresistance, both agents showed relatively higher IC_50_ values (18.5 ± 0.8 µg/mL for the extract vs. 16.7 ± 1.1 µg/mL for cisplatin), reflecting lower susceptibility of this phenotype.

These findings suggest a broad-spectrum anticancer activity of the extract, as it effectively inhibited the growth of multiple cancer cell lines from different tissue origins, including head and neck, colorectal, ovarian, and breast cancer [13]. The significant cytotoxic effects observed in ES2, SAS, and HT-29 cells, along with the moderate effect on MDA-MB-231, indicate that the extract may interfere with key oncogenic pathways shared across various cancer types. However, further investigation is needed to identify the specific molecular targets and mechanisms underlying its anticancer effects, which could provide valuable insights for future therapeutic development. Additionally, its low toxicity toward normal HDF highlights its potential selectivity for malignant cells, making it a promising candidate for further preclinical development as an anticancer agent [14]. This selective cytotoxicity may be explained by inherent differences between cancerous and normal cells. The selective cytotoxicity of the *L. nobilis* ethanolic extract toward malignant cells over normal human fibroblasts may be attributed to several cancer-specific biological characteristics. One key factor is the significantly higher proliferation rate of cancer cells compared to normal fibroblasts [15]. Rapidly dividing cells are more susceptible to agents that interfere with DNA replication, cell cycle progression, and oxidative stress—all of which are known mechanisms of action for phenolic compounds such as quercetin, kaempferol, and gallic acid identified in our extract [16]. Moreover, cancer cells typically exhibit elevated basal levels of reactive oxygen species and a disrupted redox balance [17], rendering them more vulnerable to additional oxidative stress induced by phenolic compounds [18].

### 2.2. Apoptosis Evaluation and Cell Cycle Analysis

**Figure 2:**
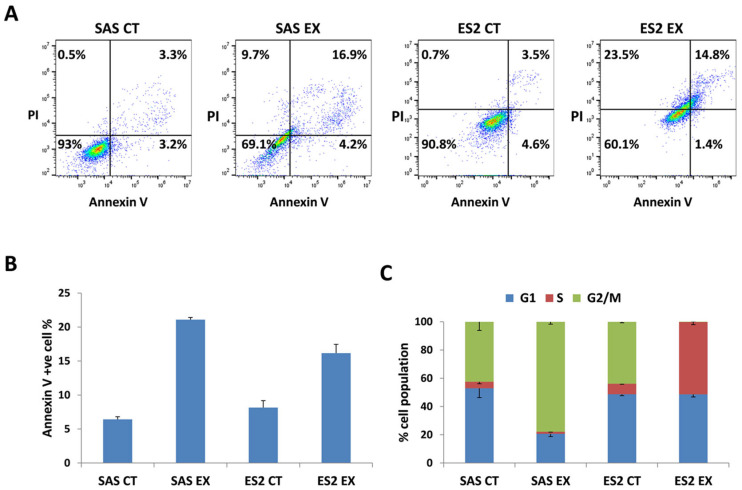
The extract induces apoptosis and cell cycle arrest in SAS and ES2 cells. Cells were treated with DMSO (CT) or the *L. nobilis* leaves ethanolic extract (EX) for 72 h. (**A**) Representative fluorescence images of different treatment groups. The *x*-axis represents Annexin V fluorescence intensity, while the *y*-axis indicates PI fluorescence intensity. (**B**) Numerical representation of Annexin +ve cells. (**C**) Cell cycle distribution of SAS and ES2 cells analyzed by flow cytometry following PI staining. The proportions of cells in G1, S, and G2/M phases are shown as percentages of the total cell population. Results in (**B**,**C**) are expressed as means and standard deviation taken from 3 independent experiments.

To further investigate the mechanisms underlying the reduced cell viability, the induction of apoptosis in SAS and ES2 cells following extract treatment was assessed using Annexin V/PI double staining, as described in Section 3 [19]. The results, presented in Figure 2A,B, reveal a significant increase in Annexin V-positive apoptotic cells after treatment with the extract (SAS: control 6.3% ± 0.4% vs. extract 21.1% ± 0.3%, *n* = 3) and (ES2: control 8.1% ± 1.0% vs. extract 16.2% ± 1.3%, *n* = 3). These findings suggest that the extract may promote cell death through apoptosis, consistent with previous observations of plant extract-induced apoptosis in cancer cells [20]. However, further investigations are required to confirm this apoptotic effect by examining caspase activation, mitochondrial membrane potential, and ROS generation, which are key markers of intrinsic apoptotic pathways [21].

To further investigate the mechanism of action of the *L. nobilis* extract, we examined its impact on cell cycle progression in SAS and ES2 cells. As shown in Figure 2C, treatment with the extract resulted in a significant accumulation of cells in the G2 phase (SAS: control 42.5% ± 6.1% vs. extract 77.9% ± 1.7%, *n* = 3). This was accompanied by a marked reduction in the proportion of cells in the G1 and S phases, indicating G2 phase arrest (G1 phase: control 52.9% ± 6.5% vs. extract 20.7% ± 1.9%, *n* = 3; S phase: control 4.6% ± 1.4% vs. extract 1.4% ± 0.2%, *n* = 3). In contrast, ES2 cells exhibited a distinct response, with extract treatment leading to a marked accumulation in the S phase (control 7.4% ± 0.3% vs. extract 51.2% ± 1.9%, *n* = 3) and a substantial reduction in the G2 phase population (control 43.9% ± 0.7% vs. extract 20.7% ± 1.9%). These findings indicate that *L. nobilis* extract induces cell cycle arrest in a cell type-dependent manner, consistent with the phase-specific responses seen in many plant-derived compounds [22,23]. The observed disruption in normal cell cycle progression, particularly G2 arrest in SAS and S phase accumulation in ES2, may prevent cancer cells from entering mitosis, thus inhibiting their proliferation and contributing to the extract’s anticancer potential [24]. This finding indicates that the extract may exert its anticancer effects by inducing cell cycle arrest at a critical checkpoint, thereby inhibiting tumor cell proliferation [25]. Further studies are needed to elucidate the molecular mechanisms underlying this effect, including the potential involvement of key cell cycle regulators such as CDK1, Cyclin B1, and WEE1 kinase. Cell cycle arrest, notably at the G2 and S phases, is often associated with the activation of apoptotic pathways, especially when cells are unable to repair damage or resume progression [26]. Here, the extract’s ability to halt cell cycle progression may also trigger apoptosis, thereby enhancing its anticancer potential. These findings suggest a dual mechanism of action, involving both cell cycle disruption and induction of programmed cell death [27].

Moreover, previous studies have evaluated various *L. nobilis* extracts for their cytotoxic potential across different cancer cell lines. Essential oils derived from *L. nobilis* leaves and seeds demonstrated weak to moderate cytotoxicity, with IC_50_ values exceeding 500 µg/mL against SH-SY5Y neuroblastoma cells [28] and 75–95 µg/mL against K562 leukemia cells [29]. In stark contrast, our phenolic-rich ethanolic leaf extract exhibited markedly stronger anticancer activity, with IC_50_ values ranging from 3.8 to 18.5 µg/mL against the investigated cell lines. This represents more than a 20-fold increase in potency compared to essential oil extracts. These findings underscore the importance of non-volatile, polyphenolic constituents—such as quercetin, kaempferol, and gallic acid—identified in our extract via UPLC-QTOF/MS and known for their pro-apoptotic and antiproliferative effects [30].

Similarly, chloroform extracts and their fractions showed lower cytotoxic activity against SK-N-BE (2)-C and SH-SY5Y neuroblastoma cell lines, with IC_50_ values ranging from 19 to 250 µg/mL [23]. A previous ethanolic extract also exhibited an IC_50_ of 24.49 µg/mL against the MCF-7 breast cancer cell line [31] This aligns with our findings against the MDA-MB-231 breast cancer cell line (IC_50_ = 18.5 ± 0.8 µg/mL), although our extract demonstrated even greater potency against SAS, ES2, and HT-29 cells. Collectively, these comparisons highlight the potential of phenolic-rich ethanolic extracts as promising sources for the development of selective anticancer agents from *L. nobilis*.

Although the *L. nobilis* extract is slightly less potent than standard chemotherapeutic agents such as cisplatin, which typically exhibit IC_50_ values of 1.6–4 µg/mL against the investigated cell lines [32], its cytotoxic activity remains remarkable for a crude plant extract. Notably, these values are substantially lower than those reported for many other botanical extracts [33].

### 2.3. Antioxidant Activity Extracts by DPPH Assay

The antioxidant activity of the *L. nobilis* leaves extract was determined by DPPH radical scavenging assay. The scavenging of DPPH radicals caused by hydrogen donation from antioxidants result in decrease DPPH radical absorbance at 517 nm [34]. As shown in Table 2, the extract showed significant concentration-dependent free radical scavenging activity from (25–2000 µg/mL), with a IC_50_ at a concentration of 713 µg/mL as compared with the standard ascorbic acid (2.6 µg/mL). Low IC_50_ values correspond to high antioxidant activity [34].

The results could hint to considerable quenching actions of phenolic compounds like flavonoids found in laurel leaves, which have been detected by LC-MS/MS, against DPPH radicals. As excessive free radicals contribute to many chronic clinical conditions, the extract may help to minimize the radicals damage [34]. This antioxidant capability is especially important in the context of oxidative stress and disease development. Antioxidants are widely characterized as “substances that delay, prevent, or remove oxidative damage to specific target molecules by reacting with oxidants,” such as ROS. Antioxidants and oxidants in equilibrium maintain redox homeostasis, while an imbalance in favor of oxidants can cause oxidative stress, which has been associated with cancer etiology. Cancer tissues are thought to produce more ROS because to altered metabolism, inflammation, a hypoxic environment, and oncogene-induced activation of ROS-generating enzymes, highlighting the role of antioxidants in buffering excess ROS and potentially slowing cancer growth [35]. Besides from redox regulation, antioxidants may also induce intrinsic cellular responses that contribute to cancer cell death. one of the most potent cytotoxic processes activated by these natural antioxidant is apoptosis. This approach can cause planned cell death and work alongside with necrosis. Because there are distinct indicators for determining which apoptotic pathway has happened, the action mechanism of each medication may be studied more precisely in future investigations [36].

### 2.4. Bioactive Compounds in L. nobilis Leaves Ethanolic Extract

In order to provide insight into the polyphenolic composition of the *L. nobilis* leaves ethanolic extract, UPLC-MS/MS analysis was performed. A number of phenolic compounds, including flavonoids, and phenolic acids, were identified in the extract Table 3.

Phenolic compounds have attracted great attention on the strength of their capacity to enhance the anticancer activity of conventional chemotherapeutic agents. There is growing evidence that suggests such bioactive compounds have the potential to exert synergistic anticancer effects in various cancer cell lines and therefore represent potential adjuncts to anticancer therapy [37].

Flavonoids are promising cytotoxic anticancer compounds due to their biological potential and minimal side effects. Flavonoids exert their anticancer effect by various mechanisms like ROS scavenging activity, suppress cancer cells proliferation, and participating in arresting the cell cycle [38]. Several key flavonoids have been identified in the *L. nobilis* leaves ethanolic extract (Table 3) like quercetin, kaempferol, naringenin, and rutin where previous studies have shown their potential role against various cancer cell lines [39] and therefore they contribute to the observed cytotoxicity of *L. nobilis* leaves extract against ES2, SAS, and HT-29 cancer cells.

Naringin, a flavonoid polyphenol found primarily in grapefruit and certain citrus fruits, has been reported to exhibit chemosensitizing activity, particularly in combination with paclitaxel. Naringin has been shown by research to enhance the cytotoxicity of paclitaxel in both androgen-independent and androgen-dependent human prostate cancer cells [40]. The enhancement procedure was performed through the mediation of intrinsic apoptosis pathway activation and the G1 cell cycle arrest. Naringin also had the ability to enhance PTEN expression, a negative regulator of the PI3K/Akt signaling cascade. In parallel, it prevents cancer cell invasion and migration through the downregulation of NF-κB, Snail, Twist, and c-Myc mRNA levels, all of which are implicated in metastasis and cancer development [41].

Similarly, quercetin, a well-studied phenolic compound, was also identified to be a potentiator of temozolomide, an oral alkylating agent employed in glioblastoma treatment. In vitro testing on U87 and U251 glioblastoma cell lines revealed that quercetin sensitize the cells to temozolomide by blocking heat-shock proteins, which are typically at the center of chemoresistance mechanisms [41].

Furthermore, in a phase I clinical trial, resveratrol dramatically decreased the levels of circulating cancer biomarkers such as Insulin-like growth factor 1 and Insulin-like growth factor-binding protein 3 [42]. Another phase I, double-blind, randomized clinical investigation found that resveratrol raised the levels of cleaved caspase-3 in malignant liver tissue [43].

Phenolic acids, a large group of non-flavonoid phenolics of plant origin, exist either in the free form (aglycones) or as glycosides. They are distributed extensively in all food sources like oilseeds, cereals, legumes, fruits and vegetables, herbs, and an enormous variety of beverages. These phenolic acids have attracted increasing attention due to their versatile biological activities, with particular interest in their potential anticancer activity [44]. The UPLC-MS/MS analysis of the *L. nobilis* leaves ethanolic extract indicated the presence of biologically important phenolic acids like gallic acid, caffeic acid, cinnamic acid, and vanillic acid. It was previously reported that phenolic acids show their anticancer effect through antioxidant activity, cell cycle arrest, and induction of apoptosis [45] which may explain the observed antioxidant and cytotoxicity of *L. nobilis* leaves extract.

Gallic acid, which is well-characterized phenolic acid, showed potential anticancer activity in vitro. Its activity was recently studied by Ko et al. against the A549 NSCLC cell line. Cell viability was studied after 48 h through a dose-dependent cell proliferation inhibition assay, suggesting its potential as a cytotoxic agent against NSCLC cells [46]. Similarly, caffeic acid has shown activity against estrogen receptor-positive (ER+) and estrogen receptor-negative (ER−) breast cancer cell lines. Rosendahl et al. established that caffeic acid is an anti-estrogenic mimic and disrupts significant signaling pathways, including the IGF-IR/pAkt and ER/cyclin D1 axes. Disruption results in cell cycle arrest and reduced proliferation, and the findings suggest that caffeic acid sensitizes tumor cells to endocrine treatment such as tamoxifen, enhancing the treatment response [47]. Vanillic acid has also been reported to have anticancer properties. In an experiment that tested its effect on breast and prostate cancer cell lines, it was determined that vanillic acid level is associated with reduced viability of cells. These results suggest that vanillic acid may be among the major compounds responsible for the antiproliferative effects in these cancer models, thus a suitable candidate as a bioactive compound to be involved in dietary cancer prevention [48].

The present study highlights a key strength compared to previous studies in that it evaluated the selective anticancer activity of the crude ethanolic extract of *L. nobilis* against different cancer cell lines, including some (e.g., SAS head and neck cancer cells) that have not been studied before, while simultaneously assessing its safety on normal fibroblasts. Unlike many earlier studies that focused primarily on general antioxidant or cytotoxic properties, this work offers evidence of both selectivity and underlying mechanisms, including apoptosis induction and cell cycle arrest. Collectively, these findings underscore the therapeutic promise of phenolic compounds in overcoming drug resistance and enhancing the anticancer efficacy of standard chemotherapeutic agents. Though the current study is limited to in vitro assays and crude extract analysis, and the samples examined were not standardized—making the reproducibility of the findings dependent on the stability of the extract’s composition—which highlights the need for further in vivo and mechanistic studies to validate and extend these results.

## 3. Materials and Methods

### 3.1. Plant Material and Extraction

The leaves of *L. nobilis* were purchased from the local traditional herbal market in Amman, Jordan. The plant was taxonomically identified with the assistance of Dr. Al-Gharaibeh, Faculty of Agriculture, Jordan Department of Plant Production, University of Science and Technology. The voucher specimens (No. Bay-2023) were deposited at the Department of Pharmaceutical Sciences, Faculty of Pharmacy, Zarqa University, Zaqa, Jordan (Pharmacognosy Lab). The ground leaves of *L. nobilis* leaves (50 g) were extracted with 70% ethanol at 60 °C in a Soxhlet apparatus until the refluxed solvent became colorless. The extract was separately evaporated to dryness in a rotary vacuum evaporator, then weighted. The ethanolic extract was stored at 4 °C until subjected to analysis and in vitro tests.

### 3.2. Cell Culture

Prof. Stephan Feller, Institute of Molecular Medicine, Martin Luther University, Germany, kindly provided the human cancer cell lines MDA-MB-231 (breast cancer cells), SAS (head and neck cancer cells), HT-29 (colorectal cancer cells), and ES2 (ovarian cancer cells). The human dermal fibroblast (HDF) cell line was obtained as a kind gift from Prof. Lina Dahabiyeh (Jordan University, Amman, Jordan). The choice of cancer cell lines was based on their clinical and epidemiological significance, given that breast, colorectal, ovarian, and head and neck cancers rank among the most prevalent malignancies in Jordan. HDFs were included as a non-malignant control to evaluate the extract’s selectivity toward cancer cells.

Cancer cell lines were cultured in Dulbecco’s Modified Eagle’s Medium (DMEM) (Sigma-Aldrich, Darmstadt, Germany) at 37 °C in a humidified incubator with 5% CO_2_. Human dermal fibroblasts were maintained under the same conditions using Iscove’s Modified Dulbecco’s Medium (IMDM (Sigma-Aldrich, Darmstadt, Germany). The media were supplemented with 10% fetal bovine serum (FBS), 100 µg/mL streptomycin, 100 U/mL penicillin, and 1% non-essential amino acids. Cell cultures were grown to confluence, with the medium replaced twice weekly.

### 3.3. In Vitro Cytotoxicity Assay

The MTT (3-[4,5-dimethylthiazol-2-yl]-2,5-diphenyl tetrazolium bromide) assay was used to examine cell viability and to calculate the half-maximal inhibitory concentration (IC_50_) values of the *L. nobilis* extract against various cancer cells and the HDF cell line. A stock solution of the extract was prepared at 100 mg/mL in DMSO, where it dissolved completely. Cells were seeded in 96-well plates at a density of 6000 cells/well for MDA-MB-231, HT-29, and HDF cells and 4000 cells/well for SAS and ES2 cells. After 24 h of incubation, cells were treated with serial concentrations of the extract or cisplatin (100, 30, 10, 3, 1, 0.3, 0.1, and 0.03 µg/mL) for 72 h. At the end of the treatment period, the supernatant was aspirated, and 50 µL of fresh DMEM containing MTT (0.5 mg/mL) was added to each well. Cells were then incubated at 37 °C for 2–4 h. The medium was then completely removed, and 50 µL of DMSO was added to dissolve the precipitated formazan crystals formed by viable cells [49].

The Synergy HTX Multimode Reader (BioTek, Shoreline, WA, USA) was used to measure cell viability, as represented by the absorbance of the formazan solution, at 570 nm. For each concentration, the relative cell viability was calculated as follows:

Relative cell viability = (mean treatment absorbance/mean control absorbance) × 100%.

Assays were performed in duplicate on three independent experiments. IC_50_ values were determined utilizing the four-parameter logistic function within the Sigmaplot 12.5 software and are expressed as the mean ± SD derived from three independent experiments.

### 3.4. Cell Cycle Analysis

SAS and ES2 cells were seeded in 6-well plates at a density of 1 × 10^5^ cells/well and treated for 72 h with the IC_50_ concentration of the extract or DMSO as a vehicle control. Following treatment, both the floating dead cells and attached cells were collected and fixed in cold ethanol at −20 °C for 30 min. Cells were then stained with a propidium iodide (PI) working solution (1 µg/mL PI and 10 µg/mL RNase A in PBS) for 30 min at 37 °C in the dark, followed by PBS washing [50]. Fluorescence was analyzed using a FACS Accuri C6 Plus flow cytometer (BD Biosciences, San Jose, CA, USA). Histograms representing cell counts versus fluorescence intensity were generated, and cell cycle distribution was determined based on mean fluorescence intensity values. A total of 10,000 events were measured in each experiment and analyzed using FlowJo software (version 10). All flow cytometry experiments were performed in three independent biological replicates. Results are reported as mean ± standard deviation (SD) to reflect reproducibility across independent experiments.

### 3.5. Apoptosis Evaluation by Annexin V-FITC/PI Staining

SAS and ES2 cells were seeded in 6-well plates and treated with the extract or DMSO as previously described. Following treatment, both floating dead cells and attached cells were collected, washed with PBS, and resuspended in 100 μL of Annexin V solution for 30 min, following the manufacturer’s instructions (Miltenyi Biotec, Bergisch Gladbach, Germany) #130-092-052) [51]. After Annexin V staining, cells were briefly stained with PI to exclude dead cells. Apoptosis quantification was performed using a FACS Accuri C6 Plus flow cytometer (BD Biosciences, San Jose, CA, USA), with 10,000 events recorded and analyzed using FlowJo software (version 10).

### 3.6. Antioxidant Assay

The free radical scavenging activity of *L. nobilis* leaves extract was determined by measuring the decrease in absorbance of DPPH at 517 nm as described by Gulcin et al. (2003) [52]. Briefly, 1 mL of 0.1 mM DPPH in methanol was added to 3 mL of bay extract solution in methanol at various concentrations (25–2000 µg/mL). The resulting mixture was incubated in the dark for 30 min, and then absorbance was measured at 517 nm. The control sample was prepared by adding 1 mL of DPPH to 3 mL of methanol. Vitamin C was used as a reference standard.

The ability of the extract to scavenge DPPH radical was calculated according to the following equation:

DPPH Scavenging Activity = [(Ao − A1/Ao) × 100]

where Ao is the absorbance of the control reaction and A1 is the absorbance in the presence of the sample of *L. nobilis* extract. The results were expressed as mean value ± standard deviation (SD).

### 3.7. Identification of Bioactive Compounds in L. nobilis Leaves Ethanolic Extract

Ultra-performance liquid chromatography (UPLC) equipped with quadrupole time-of-flight mass spectrometry (Q-TOF/MS) was utilized to characterize bioactive chemicals. The UPLC chromatographic conditions are detailed in Table 4. The Apollo II ion funnel electrospray source was employed for ionization in both positive and negative modes. The mass spectrometry conditions were as follows:

- Capillary voltage: 2500 volts (VT);
- Nebulizer gas pressure: 2.0 bar;
- Dry gas (nitrogen) flow rate: 8 L/min;
- Dry gas temperature: 200 °C.

The instrument provided a mass resolution of 50,000 full sensitivity resolution (FSR) and a mass accuracy of 1 ppm. The time-of-flight (TOF) repetition rate reached up to 20 kHz. Data analysis was conducted using the Data Analysis 4.2 software (Bruker Daltonics, Bremen, Germany). Compound identification was carried out by LC-MS/MS using two complementary approaches: (i) direct comparison with authentic standards when available, and (ii) tentative assignment based on accurate mass and fragmentation patterns with spectral library matching.

## 4. Conclusions

*L. nobilis* leaf extract exhibited selective anticancer activity against ovarian (ES2), head and neck (SAS), and colorectal (HT-29) cancer cell lines, while showing minimal cytotoxicity toward normal fibroblasts. Its effects included the induction of apoptosis and interruption of cancer cell proliferation by causing G2 phase arrest in SAS cells and S phase accumulation in ES2 cells. The extract was also found to have antioxidant activity, though less than ascorbic acid. These effects can be attributed to the known phenolic compounds such as flavonoids and phenolic acids.

Notably, the crude ethanolic extract (10% yield from dried leaves) was directly assessed without additional purification, which may pose challenges in pinpointing the exact bioactive molecules. Nevertheless, the LC-MS chromatogram of the crude extract supports the presence of diverse phenolic constituents, likely contributing synergistically to the observed effects. Future studies involving fractionation, purification, and detailed profiling are warranted to improve reproducibility and to detect the compounds most responsible for the anticancer and antioxidant activities.

Based on this study, *L. nobilis* may be deemed to possess excellent potential as a source of natural development of selective anticancer and antioxidant drugs and deserves further research.

## Figures and Tables

**Table 1:** IC_50_ values of the *L. nobilis* extract and cisplatin against cancer cell lines and normal fibroblasts (µg/mL).

Cell Line	*L. nobilis* Extract	Cisplatin
ES2	4.2 ± 0.2	3.6 ± 0.3
SAS	3.8 ± 0.3	2.9 ± 0.5
HT-29	4.4 ± 0.6	10.7 ± 2.1
MDA-MB-231	18.5 ± 0.8	16.7 ± 1.1
HDF	>100	>100

**Table 2:** DPPH scavenging activity of *L. nobilis* leaves ethanolic extract.

Concentration (µg/mL)	DPPH Scavenging Activity %
25	4.2 ± 0.002
50	8.9 ± 0.001
100	15.7 ± 0.0006
200	27.2 ± 0.002
500	41.9 ± 0.002
1000	55.5 ± 0.0006
2000	77.5 ± 0.0006

**Table 3:** Phenolic compounds and their phytochemical class identification in *L. nobilis* leaves ethanolic extract using the LC-MS/MS.

N	RT (min)	Identified Compound Name	Phytochemical Class	Chemical Formula	Molecular Weight (Da)	*m/z*	Ion Mode
1	3.39	Gallic acid	Phenolic acid	C_7_H_6_O_5_	170.0216	171.0289	[M+H]^+^
2	5.29	Quercetin	Flavonoid	C_15_H_10_O_7_	302.04226	303.04954	[M+H]^+^
3	5.35	Isorhamnetin	Flavonoid	C_16_H_12_O_7_	316.05796	317.06524	[M+H]^+^
4	5.8	Kaempferol	Flavonoid	C_15_H_10_O_6_	286.04752	287.0548	[M+H]^+^
5	6.24	Resveratrol	Stilbenoid	C_14_H_12_O_3_	228.07855	229.08582	[M+H]^+^
6	11.15	4-hydroxycoumarin	Phenolic acid	C_9_H_6_O_3_	162.0317	163.03894	[M+H]^+^
7	1.52	Anisic acid	Phenolic acid	C_8_H_8_O_3_	152.0474	153.05472	[M+H]^+^
8	1.89	Gallocatechin	Flavonoid	C_15_H_14_O_7_	306.07196	307.07923	[M+H]^+^
9	3.07	Caffeic Acid	Phenolic acid	C_9_H_8_O_4_	180.0399	181.04718	[M+H]^+^
10	3.17	Chlorogenic acid	Phenolic acid	C_16_H_18_O_9_	354.09439	355.10166	[M+H]^+^
11	3.98	o-Coumaric acid	Phenolic acid	C_9_H_8_O_3_	164.04732	165.0546	[M+H]^+^
12	4.24	Catechin	Flavonoid	C_15_H_14_O_6_	290.07941	291.08652	[M+H]^+^
13	4.25	Naringenin	Flavonoid	C_15_H_12_O_5_	272.06789	273.07516	[M+H]^+^
14	4.51	Vanillic acid	Phenolic acid	C_8_H_8_O_4_	168.04219	169.04947	[M+H]^+^
15	4.74	Scopoletin	Coumarin	C_10_H_8_O_4_	192.04185	193.04913	[M+H]^+^
16	4.82	Vitexin	Flavonoid	C_21_H_20_O_10_	432.10515	433.11243	[M+H]^+^
17	4.91	Rutin	Flavonoid	C_27_H_30_O_16_	610.15234	611.1598	[M+H]^+^
18	4.96	Hyperoside	Flavonoid	C_21_H_20_O_12_	464.0949	465.10217	[M+H]^+^
19	5.28	Saponarin	Flavonoid	C_27_H_30_O_15_	594.1577	595.16512	[M+H]^+^
20	6.51	Cinnamic acid	Phenolic acid	C_9_H_8_O_2_	148.05233	149.05961	[M+H]^+^
21	3.82	ProcyanidinB2	Flavonoid	C_30_H_26_O_12_	578.14289	577.13561	[M−H]^−^

The identified chemicals were compared with some existing literature on anticancer mechanisms. The findings are elaborated in Supplementary Table S1.

**Table 4:** Summary of chromatographic conditions.

UPLC Conditions	Injection Volume	Flow Rate	Autosampler Temperature	Column Oven Temperature	Total Run Time
	3 µL	0.51 mL/min	8 °C	40 °C	35 min.
Chromatography	Mobile phase	Solvents: (A) Water with 0.1% methanol (B) Methanol 0–3 min, 5% B; 3–23 min, 60% B; 23–28 min, 95% B; 28–31 min, 95% B; 31.0–31.1 min, 5% B; 31.1–35 min 5% B;			
	Column type	Bruker Daltonik (Berlin, Germany) C-18 column (100 × 2.1 mm × 2 µm) (120 Aº)			

## Data Availability

The original contributions presented in this study are included in the article. Further inquiries can be directed to the corresponding author.

## Supplementary Materials

The following supporting information can be downloaded at: https://www.mdpi.com/article/10.3390/molecules30194012/s1, Table S1: Some of previously reported anticancer activities of identified phenolic compounds in *Laurus nobilis* ethanolic leaf extract; Figure S1: Chromatograms of compounds identified in *L. noblis* extract, see [53,54,55,56,57,58,59,60,61,62,63,64,65,66,67,68,69,70,71,72,73,74,75,76,77,78,79,80,81,82,83,84,85,86,87,88,89,90,91,92,93,94,95,96,97,98,99,100,101,102,103,104,105,106,107,108,109,110,111].

## Abbreviations

The following abbreviations are used in this manuscript:

DMSO	Dimethyl Sulfoxide
DMEM	Dulbecco’s Modified Eagle’s Medium
FBS	Fetal Bovine Serum
FSR	Full Sensitivity Resolution
IC_50_	Half Maximal Inhibitory Concentration
HDF	Human Dermal Fibroblast
IGF-IR	Insulin-like Growth Factor-1 Receptor
IMDM	Iscove’s Modified Medium
NSCLC	Non-Small Cell Lung Cancer
NF-ĸB	Nuclear Factor KappaB
PTEN	Phosphate and TENsin Homolog
PBS	Phosphate-Buffer Saline
PI3K	Phosphoinositide 3-Kinases
PI	Propidium Iodide
Q-TOF/MS	Quadrupole Time-of-Flight Mass Spectrometry
ROS	Reactive Oxygen Species
TOF	Time-of-Flight
UPLC	Ultra Performance Liquid Chromatography
