# Supplementary material for: The Therapeutic Potential of Laurus nobilis L. Leaves Ethanolic Extract in Cancer Therapy
Source: Molecules. 2025 Oct 7;30(19):4012. doi: 10.3390/molecules30194012 (PMC12526220; doi:10.3390/molecules30194012)
Supplement: Supplementary file 1 [file molecules-30-04012-s001.zip › molecules-3838029-supplementary.pdf]

Supplementary Table S1. Some of previously reported anticancer activities of identified phenolic compounds in *Laurus nobilis* ethanolic leaf extract.

Identified compound name	Chemical structure	Reported anti-cancer mechanism	Cancer types	Referenses
Gallic acid	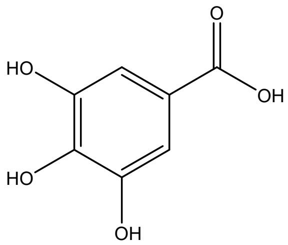	Cancer cell proliferation inhibition, apoptosis induction and cell cycle arrest.	NSCLC, prostate cancer, osteosarcoma, leukemia, colon cancer, glioblastoma, pancreatic cancer, oral cancer, melanoma, breast cancer, cervical cancer and skin cancer	[1-3]
Quercetin	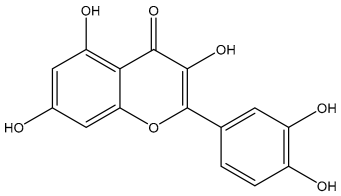	Cancer cell proliferation inhibition, apoptosis induction, inhibitin g angeogenesis, modulation of PI3K/Akt/mTOR, Wnt/β-catenin, and MAPK/ERK1/2 pathways and cell cycle arrest.	Lymphoma, ovarian cancer, breast cancer, gastric cancer, osteosarcoma, prostate cancer and leukemia , colon cancer	[4-7]
Isorhamnetin	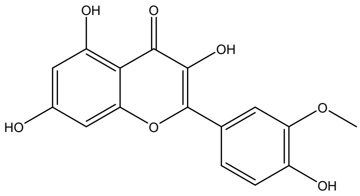	Induces ROS-mediated AMPK signaling; downregulates MEK1, PI3K, NF-κB, PI3K/AKT/mTOR and Akt/ERK and inhibition of Hypoxia Inducible Factor (HIF)-1α expression.	Colon cancer, skin cancer, brest cancer, pncreatic cancer, bladder cancer, gastric cancer, melanoma, leukemia	[8-11]
Kaempferol	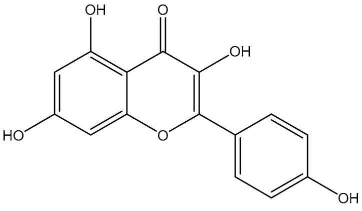	Cell cycle arrest, downregulation of CDK1, oxidatice damage mediated apoptosis, and EMT inhibition	Breast cancer, bladder cancer, prostate cancer, cervical cancer, liver cancer, ovarian cancer, leukemia,	[12-15]

Resveratrol	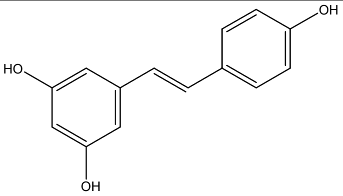	Apoptosis, cell cycle arest.	Colon cancer, prostate cancer, breast cancer, ovarian cancer, lung cancer, kidny cancer, liver cancer	[16,17]
4-hydroxycoumarin	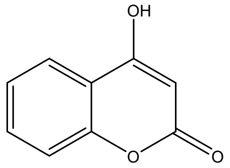	Apoptosis and cell cycle arrest,.	Liver cancer, colon cancer, breast cancer	[18,19]
Anisic acid	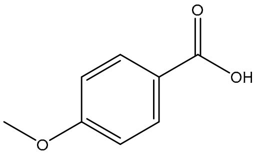	Cancer cell proliferation inhibition and apoptosis.	Leukemia, breast cancer, colon cancer, melanoma	[20,21]
Gallocatechin	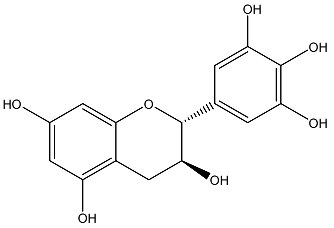	Anti-oxidant.	Limited anti-cancer data	-----
Caffeic Acid	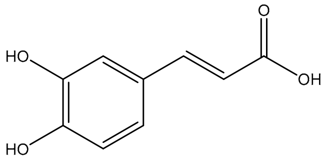	Prevent production of reactive oxygen species, DNA damage and increse apoptosis.	Uterine cervix cancer, liver cancer, breast cancer, lung cancer, melanoma, oral cancer, colorectal cancer	[22-25]
Chlorogenic acid	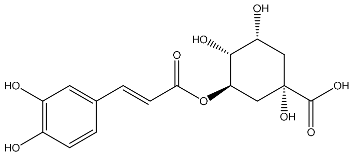	Cancer cell proliferation inhibition, inhibit mitochondreal ATP production and antioxidant.	Lung cancer, liver cancer, cervical cancer, kidney cancer, colon cancer, glioblaastoma, breast cancer	[26-29]
o-Coumaric acid	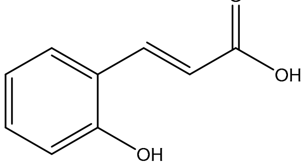	Anti-proliferative effect, reduce cell viabality and apoptotic effect.	Lung cancer, glioblastoma, breast cancer	[30-32]

Catechin	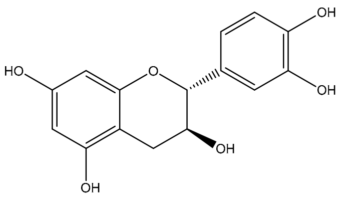	Cell cycle arrest and cell apoptosis and activation of ROS mediated mitochondrial apoptotic pathways.	Breast cancer, lung cancer, prostate cancer, colorectal cancer, pancreatic cancer	[33-35]
Naringenin	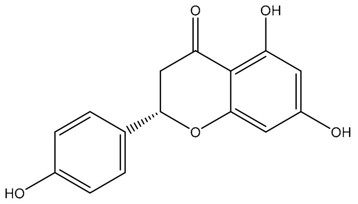	Cell cycle arrest, apoptosis, blocking of the PI3K/AKT pathway and activation of pro-death autophagy.	Breast cancer, lung cancer, gastric cancer, colorectal cancer, prostate cancer, melanoma	[36-38]
Vanillic acid	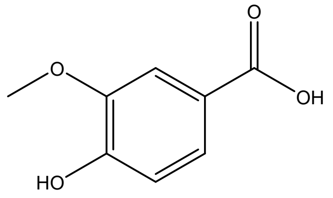	Enhance the expression of pro-apoptotic proteins, decrease gene expression of Bcl-2, angiogenesis inhibition and cell cycle arrest.	Lung cancer, colon cancer	[39-42]
Scopoletin	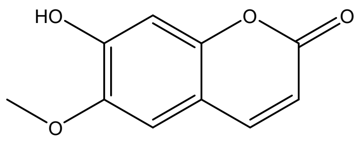	Apoptotic cell death, decrease gene expression of Bcl-2 and mitochondreal depolarization and apoptosis.	Cervical cancer, lung cancer, breast cancer	[43-45]
Vitexin	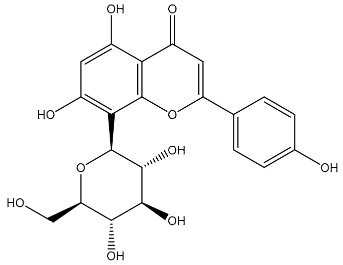	Apoptosis, reduction of p-PI3K, p-Akt and p-mTOR levels, reduction of cell viability.	Lung cancer, cervical cancer, breast cancer	[46-48]

Rutin	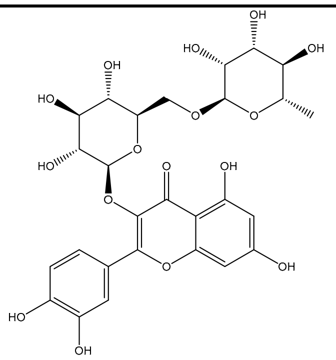	Decreas cell viability, anti-proliferative effect and anti-apoptotic effect.	Renal cancer, lung cancer,	[49,50]
Hyperoside	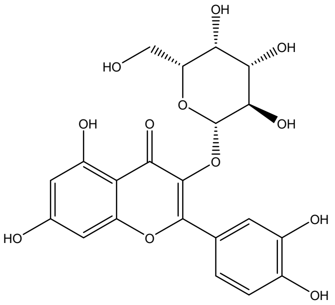	Inhibit proliferation, induce apoptosis and autophagy and apoptosis by ROS mediated NF-kB signaling pathway.	Skin cancer, breast cancer, lung cancer	[51-53]
Saponarin	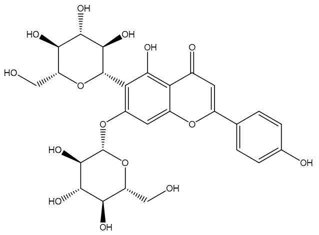	Anti-inflamatory.	Limited anti-cancer data	----
Cinnamic acid	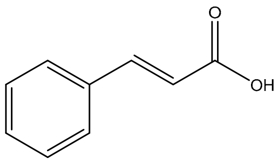	Cell cycle rrest, apoptosis and inhibition of cancer cell growth.	Breast cancer, leukemia, prostate cancer	[54-56]
Procyanidin B2	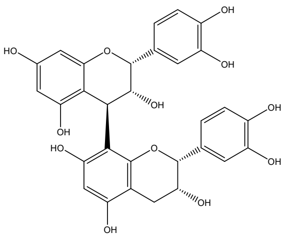	Apoptosis, autophagy and decrease cell viability.	Colorectal cancer, gastric cancer, breast cancer	[57-59]

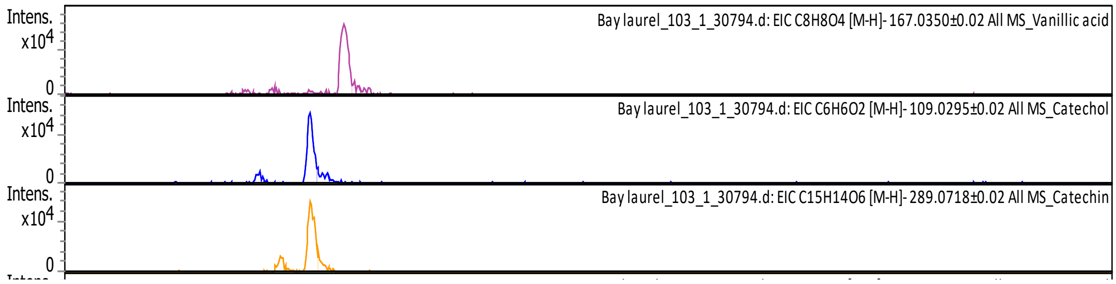

Figure S1: Chromatograms of compounds identified in *L.noblis extract*

References:

1. Zhang T, Ma L, Wu P *et al.* Gallic acid has anticancer activity and enhances the anticancer effects of cisplatin in non-small cell lung cancer A549 cells via the JAK/STAT3 signaling pathway. *Oncology reports*, 41(3), 1779-1788 (2019).
2. Jiang Y, Pei J, Zheng Y, Miao Y-j, Duan B-z, Huang L-f. Gallic acid: A potential anti-cancer agent. *Chinese journal of integrative medicine*, 28(7), 661-671 (2022).
3. Aborehab NM, Osama N. Effect of Gallic acid in potentiating chemotherapeutic effect of Paclitaxel in HeLa cervical cancer cells. *Cancer cell international*, 19, 1-13 (2019).
4. Tang S-M, Deng X-T, Zhou J, Li Q-P, Ge X-X, Miao L. Pharmacological basis and new insights of quercetin action in respect to its anti-cancer effects. *Biomedicine & Pharmacotherapy*, 121, 109604 (2020).
5. Reyes-Farias M, Carrasco-Pozo C. The anti-cancer effect of quercetin: molecular implications in cancer metabolism. *International journal of molecular sciences*, 20(13), 3177 (2019).
6. Hashemzaei M, Delarami Far A, Yari A *et al.* Anticancer and apoptosis-inducing effects of quercetin in vitro and in vivo. *Oncology reports*, 38(2), 819-828 (2017).
7. Bhatiya M, Pathak S, Jothimani G, Duttaroy AK, Banerjee A. A comprehensive study on the anti-cancer effects of quercetin and its epigenetic modifications in arresting progression of colon cancer cell proliferation. *Archivum immunologiae et therapiae experimentalis*, 71(1), 6 (2023).
8. Biswas P, Kaium MA, Tareq MMI *et al.* The experimental significance of isorhamnetin as an effective therapeutic option for cancer: A comprehensive analysis. *Biomedicine & Pharmacotherapy*, 176, 116860 (2024).
9. Mei C, Liu Y, Lyu X *et al.* Advances in Isorhamnetin Treatment of Malignant Tumors: Mechanisms and Applications. *Nutrients*, 17(11), 1853 (2025).
10. Khaled R. Biological activities of isorhamnetin: A review. *Plantae Scientia*, 3(5), 78-81 (2020).
11. Park C, Cha H-J, Choi EO *et al.* Isorhamnetin induces cell cycle arrest and apoptosis via reactive oxygen species-mediated AMP-activated protein kinase signaling pathway activation in human bladder cancer cells. *Cancers*, 11(10), 1494 (2019).
12. Wang X, Yang Y, An Y, Fang G. The mechanism of anticancer action and potential clinical use of kaempferol in the treatment of breast cancer. *Biomedicine & Pharmacotherapy*, 117, 109086 (2019).
13. Imran M, Salehi B, Sharifi-Rad J *et al.* Kaempferol: A key emphasis to its anticancer potential. *Molecules*, 24(12), 2277 (2019).
14. Kaur S, Mendonca P, Soliman KF. The Anticancer Effects and Therapeutic Potential of Kaempferol in Triple-Negative Breast Cancer. *Nutrients*, 16(15), 2392 (2024).
15. Alyami NM, Alyami HM, Almeer R. Using green biosynthesized kaempferol-coated sliver nanoparticles to inhibit cancer cells growth: An in vitro study using hepatocellular carcinoma (HepG2). *Cancer Nanotechnology*, 13(1), 26 (2022).
16. Huminiecki L, Horbańczuk J. The functional genomic studies of resveratrol in respect to its anti-cancer effects. *Biotechnology Advances*, 36(6), 1699-1708 (2018).
17. Varoni EM, Lo Faro AF, Sharifi-Rad J, Iriti M. Anticancer molecular mechanisms of resveratrol. *Frontiers in nutrition*, 3, 8 (2016).
18. Öner Ç, Soyergin D, Özyurt A, Çolak E. 4-Hydroxycoumarin effects on both cellular and genetic characteristics of hepatocellular carcinoma cells. *Cytology and Genetics*, 56(3), 292-300 (2022).
19. Dimić DS, Kaluđerović GN, Avdović EH *et al.* Synthesis, Crystallographic, quantum chemical, antitumor, and molecular docking/dynamic studies of 4-hydroxycoumarin-neurotransmitter derivatives. *International journal of molecular sciences*, 23(2), 1001 (2022).
20. Czarnecka M, Świtalska M, Wietrzyk J, Maciejewska G, Gliszczyńska A. Synthesis, characterization, and in vitro cancer cell growth inhibition evaluation of novel phosphatidylcholines with anisic and veratric acids. *Molecules*, 23(8), 2022 (2018).

21. Palko-Łabuz A, Gliszczyńska A, Skonieczna M, Poła A, Wesołowska O, Środa-Pomianek K. Conjugation with phospholipids as a modification increasing anticancer activity of phenolic acids in metastatic melanoma—In vitro and in silico studies. *International Journal of Molecular Sciences*, 22(16), 8397 (2021).
22. Kanimozhi G, Prasad N. Anticancer effect of caffeic acid on human cervical cancer cells. In: *Coffee in health and disease prevention.* (Elsevier, 2015) 655-661.
23. Espíndola KMM, Ferreira RG, Narvaez LEM *et al.* Chemical and pharmacological aspects of caffeic acid and its activity in hepatocarcinoma. *Frontiers in oncology*, 9, 541 (2019).
24. Alam M, Ahmed S, Elasbali AM *et al.* Therapeutic implications of caffeic acid in cancer and neurological diseases. *Frontiers in oncology*, 12, 860508 (2022).
25. Pelinson LP, Assmann CE, Palma TV *et al.* Antiproliferative and apoptotic effects of caffeic acid on SK-Mel-28 human melanoma cancer cells. *Molecular biology reports*, 46, 2085-2092 (2019).
26. Huang S, Wang L-L, Xue N-N *et al.* Chlorogenic acid effectively treats cancers through induction of cancer cell differentiation. *Theranostics*, 9(23), 6745 (2019).
27. Yamagata K, Izawa Y, Onodera D, Tagami M. Chlorogenic acid regulates apoptosis and stem cell marker-related gene expression in A549 human lung cancer cells. *Molecular and cellular biochemistry*, 441, 9-19 (2018).
28. Bender O, Atalay A. Polyphenol chlorogenic acid, antioxidant profile, and breast cancer. In: *Cancer.* (Elsevier, 2021) 311-321.
29. Wang L, Pan X, Jiang L *et al.* The biological activity mechanism of chlorogenic acid and its applications in food industry: A review. *Frontiers in Nutrition*, 9, 943911 (2022).
30. Mutlu D. Cytotoxic activity, anti-migration and in silico study of o-coumaric acid on H1975 non-small cell lung cancer cells. *Cumhuriyet Science Journal*, 46(1), 35-40 (2025).
31. Mercado YG, Díaz JM, Hernández DO *et al.* Ortho-coumaric acid derivatives with therapeutic potential in a three-dimensional culture of the immortalised U-138 MG glioblastoma multiforme cell line. *Neurology Perspectives*, 2, S19-S30 (2022).
32. Burhanoğlu T, Halbutoğulları ZS, Turhal G, Demiroglu-Zergeroglu A. Evaluation of the anticancer effects of hydroxycinnamic acid isomers on breast cancer stem cells. *Medical Oncology*, 42(3), 73 (2025).
33. Cheng Z, Zhang Z, Han Y *et al.* A review on anti-cancer effect of green tea catechins. *Journal of Functional Foods*, 74, 104172 (2020).
34. Sun H, Yin M, Hao D, Shen Y. Anti-cancer activity of catechin against A549 lung carcinoma cells by induction of cyclin kinase inhibitor P21 and suppression of cyclin E1 and P–AKT. *Applied Sciences*, 10(6), 2065 (2020).
35. Silva C, Correia-Branco A, Andrade N *et al.* Selective pro-apoptotic and antimigratory effects of polyphenol complex catechin: lysine 1: 2 in breast, pancreatic and colorectal cancer cell lines. *European Journal of Pharmacology*, 859, 172533 (2019).
36. Stabrauskiene J, Kopustinskiene DM, Lazauskas R, Bernatoniene J. Naringin and naringenin: Their mechanisms of action and the potential anticancer activities. *Biomedicines*, 10(7), 1686 (2022).
37. Rauf A, Shariati MA, Imran M *et al.* Comprehensive review on naringenin and naringin polyphenols as a potent anticancer agent. *Environmental Science and Pollution Research*, 29(21), 31025-31041 (2022).
38. Choi J, Lee D-H, Jang H, Park S-Y, Seol J-W. Naringenin exerts anticancer effects by inducing tumor cell death and inhibiting angiogenesis in malignant melanoma. *International journal of medical sciences*, 17(18), 3049 (2020).
39. Venkidasamy B, Subramanian U, Almoallim HS, Alharbi SA, Lakshmikumar RRC, Thiruvengadam M. Vanillic Acid Nanocomposite: Synthesis, Characterization Analysis, Antimicrobial, and Anticancer Potentials. *Molecules*, 29(13), 3098 (2024).

40. Gong J, Zhou S, Yang S. Vanillic acid suppresses HIF-1α expression via inhibition of mTOR/p70S6K/4E-BP1 and Raf/MEK/ERK pathways in human colon cancer HCT116 cells. *International journal of molecular sciences*, 20(3), 465 (2019).
41. Kaur J, Gulati M, Gowthamarajan K *et al.* Combination therapy of vanillic acid and oxaliplatin co-loaded in polysaccharide based functionalized polymeric micelles could offer effective treatment for colon cancer: A hypothesis. *Medical Hypotheses*, 156, 110679 (2021).
42. Velli SK, Sundaram J, Murugan M, Balaraman G, Thiruvengadam D. Protective effect of vanillic acid against benzo (a) pyrene induced lung cancer in Swiss albino mice. *Journal of biochemical and molecular toxicology*, 33(10), e22382 (2019).
43. Tian Q, Wang L, Sun X, Zeng F, Pan Q, Xue M. Scopoletin exerts anticancer effects on human cervical cancer cell lines by triggering apoptosis, cell cycle arrest, inhibition of cell invasion and PI3K/AKT signalling pathway. *J BUON*, 24(3), 997-1002 (2019).
44. Yuan C, Wang M-H, Wang F *et al.* Network pharmacology and molecular docking reveal the mechanism of Scopoletin against non-small cell lung cancer. *Life sciences*, 270, 119105 (2021).
45. Shi Z, Chen L, Sun J. Novel scopoletin derivatives kill cancer cells by inducing mitochondrial depolarization and apoptosis. *Anti-Cancer Agents in Medicinal Chemistry (Formerly Current Medicinal Chemistry-Anti-Cancer Agents)*, 21(14), 1774-1782 (2021).
46. Liu X, Jiang Q, Liu H, Luo S. Vitexin induces apoptosis through mitochondrial pathway and PI3K/Akt/mTOR signaling in human non-small cell lung cancer A549 cells. *Biological research*, 52, 1-7 (2019).
47. Wang Q, Zhang J, Ye J, Guo J. Vitexin exerts anti-tumor and anti-angiogensis effects on cervical cancer through VEGFA/VEGFR2 pathway. *European Journal of Gynaecological Oncology*, 43(4) (2022).
48. Ghazy E, Taghi HS. The autophagy-inducing mechanisms of vitexin, cinobufacini, and physalis alkekengi hydroalcoholic extract against breast cancer in vitro and in vivo. *Journal of gastrointestinal cancer*, 1-5 (2022).
49. Caparica R, Júlio A, Araújo MEM *et al.* Anticancer activity of rutin and its combination with ionic liquids on renal cells. *Biomolecules*, 10(2), 233 (2020).
50. Paudel KR, Wadhwa R, Tew XN *et al.* Rutin loaded liquid crystalline nanoparticles inhibit non-small cell lung cancer proliferation and migration in vitro. *Life sciences*, 276, 119436 (2021).
51. Kong Y, Sun W, Wu P. Hyperoside exerts potent anticancer activity in skin cancer. *Frontiers in Bioscience-Landmark*, 25(3), 463-479 (2020).
52. Qiu J, Zhang T, Zhu X *et al.* Hyperoside induces breast cancer cells apoptosis via ROS-mediated NF-κB signaling pathway. *International Journal of Molecular Sciences*, 21(1), 131 (2019).
53. Hu Z, Zhao P, Xu H. Hyperoside exhibits anticancer activity in non-small cell lung cancer cells with T790M mutations by upregulating FoxO1 via CCAT1. *Oncology Reports*, 43(2), 617-624 (2020).
54. Hunke M, Martinez W, Kashyap A, Bokoskie T, Pattabiraman M, Chandra S. Antineoplastic actions of cinnamic acids and their dimers in breast cancer cells: a comparative study. *Anticancer research*, 38(8), 4469-4474 (2018).
55. Yenigül M, Akçok İ, Gencer Akçok EB. Ethacrynic acid and cinnamic acid combination exhibits selective anticancer effects on K562 chronic myeloid leukemia cells. *Molecular Biology Reports*, 49(8), 7521-7530 (2022).
56. Imai M, Yokoe H, Tsubuki M, Takahashi N. Growth inhibition of human breast and prostate cancer cells by cinnamic acid derivatives and their mechanism of action. *Biological and Pharmaceutical Bulletin*, 42(7), 1134-1139 (2019).

57. Zhang R, Yu Q, Lu W *et al.* Grape seed procyanidin B2 promotes the autophagy and apoptosis in colorectal cancer cells via regulating PI3K/Akt signaling pathway. *OncoTargets and therapy*, 4109-4118 (2019).
58. Li Y, Lu X, Tian P, Wang K, Shi J. Procyanidin B2 induces apoptosis and autophagy in gastric cancer cells by inhibiting Akt/mTOR signaling pathway. *BMC Complementary Medicine and Therapies*, 21, 1-9 (2021).
59. Xue H-k, Tan J-q, Li Q, Tang J-t. Procyanidin B2-induced apoptosis in MCF-7 cells and its mechanism. (2021).
